# Enhancement of Late Successional Plants on Ex-Arable Land by Soil Inoculations

**DOI:** 10.1371/journal.pone.0021943

**Published:** 2011-07-08

**Authors:** Vanesa Carbajo, Bowy den Braber, Wim H. van der Putten, Gerlinde B. De Deyn

**Affiliations:** 1 Department of Terrestrial Ecology, Netherlands Institute of Ecology, Wageningen, The Netherlands; 2 Department of Ecology, Alcalá University, Madrid, Spain; 3 Nature Conservation and Plant Ecology Group, Wageningen University and Research Centre, Wageningen, The Netherlands; 4 Laboratory of Nematology, Wageningen University and Research Centre, Wageningen, The Netherlands; University of Tartu, Estonia

## Abstract

Restoration of species-rich grasslands on ex-arable land can help the conservation of biodiversity but faces three big challenges: absence of target plant propagules, high residual soil fertility and restoration of soil communities. Seed additions and top soil removal can solve some of these constraints, but restoring beneficial biotic soil conditions remains a challenge. Here we test the hypotheses that inoculation of soil from late secondary succession grasslands in arable receptor soil enhances performance of late successional plants, especially after top soil removal but pending on the added dose. To test this we grew mixtures of late successional plants in arable top (organic) soil or in underlying mineral soil mixed with donor soil in small or large proportions. Donor soils were collected from different grasslands that had been under restoration for 5 to 41 years, or from semi-natural grassland that has not been used intensively. Donor soil addition, especially when collected from older restoration sites, increased plant community biomass without altering its evenness. In contrast, addition of soil from semi-natural grassland promoted plant community evenness, and hence its diversity, but reduced community biomass. Effects of donor soil additions were stronger in mineral than in organic soil and larger with bigger proportions added. The variation in plant community composition was explained best by the abundances of nematodes, ergosterol concentration and soil pH. We show that in controlled conditions inoculation of soil from secondary succession grassland into ex-arable land can strongly promote target plant species, and that the role of soil biota in promoting target plant species is greatest when added after top soil removal. Together our results point out that transplantation of later secondary succession soil can promote grassland restoration on ex-arable land.

## Introduction

During the last century, in industrialized countries, species-rich grasslands have become rare due to land-use intensification and atmospheric deposition of nitrogen [1], [2]. These changes have promoted a select number of high productive plant species, causing the decline of many slow growing plant species that typify species rich grasslands [3], [4]. In order to counteract this decline a fraction of the arable land is being restored into semi-natural species-rich grasslands [5]. The (re)creation of these species-rich systems, however, requires the presence of specific abiotic and biotic conditions [5]–[7]. Even after re-establishment of meso- or eutrophic systems, conditions often remain favorable for early successional, fast growing species, whereas conditions are less conducive for late successional species because of high residual fertility and N deposition [1], [4], [8]. In order to overcome this constraint of excessive soil fertility, managers mow and remove hay [6], introduce herbivores that graze and concentrate nutrients [9], add carbon rich substrates which stimulates nutrient immobilisation by soil microbes [10], [11] or they remove the entire top soil [5], [7], [12].

The potential biotic constraints for biodiversity restoration are manifold, but to date the aspect of availability of species of target plant communities received most attention. The absence of late successional species from the seed bank and poor dispersal and colonization possibilities due to habitat fragmentation can clearly impede restoration of target plant communities [13], [14]. To overcome the limitation of absence of propagules of target plant species seed additions or spreading of hay containing seeds of desired plant species can be considered [15]–[17]. However, the availability of seeds of target plant species does not guarantee their establishment and there is growing awareness that also biotic soil properties may be of key importance for vegetation, and more general, for biodiversity restoration [18], [19].

Theoretical and empirical studies show that soil biota can strongly affect the establishment, diversity and successional replacement of plant species in time series of land abandonment on grassland [20], [21] and arable land [20], [22], [23]. Soil communities consist of biota that can directly promote (e.g. mycorrhizal fungi) or suppress (e.g. root herbivores and pathogens) plant growth, and of biota that mediate these direct interactions by predation or influencing nutrient availability [24]. Compositions of soil communities are dynamic and change along secondary succession gradients. For example, bacterial biomass and abundances of plant-feeding nematodes tend to decrease and abundances of saprophytic and mycorrhizal fungi, as well as of omni- and carnivorous nematodes tend to increase after land abandonment [23], [25]–[27]. Given these transitions in soil communities it is crucial to determine whether and how the origin of soil biota, in relation to restoration history, matters for the promotion of late successional vegetation.

Impact of soil biota on plant communities is dependent on soil nutrient status [28], even to the extend that mutualism can turn into parasitism as frequently reported for mycorrhizal fungi at high soil P availability [29]–[31]. Plant growth promotion of late successional plants through soil inoculations is therefore more likely to occur in nutrient poor soil, such as soil after top soil removal, than in nutrient rich top soil. Moreover stimulation of plant growth by symbiotic soil biota is often larger when whole and diverse communities rather then when only specific taxa are used as inoculum [31]. In top soil the establishment of such introduced soil biota may be difficult given the high abundances of residing soil biota, so that new introductions might be more successful after top soil removal. Moreover the donor soil not only serves as inoculum source but it is also a good habitat for the desired soil biota so that the effect of soil inoculation is likely to increase with larger inoculum density.

Here we experimentally test whether soil inoculation could be a tool to improve restoration management strategies to restore species-rich grasslands. We tested three specific hypotheses: (1) the introduction of donor soil to promote target plant species is more successful after removal of the top layer of the arable receptor soil (2) donor soil from late successional or semi-natural grassland promotes late successional plants more than donor soil from early successional grassland (3) the impact of donor soil is dependent on dosage. We test these hypotheses under controlled conditions in a greenhouse in order to establish a proof of principle. In the case soil inoculation would work, those conditions may be studied in more detail under semi-natural and natural conditions in outdoor mesocosms and in the field.

## Results

### Effects of donor and receptor soil on plant community biomass

#### Addition of donor soil in a 1∶1 proportion

**Figure 1:**
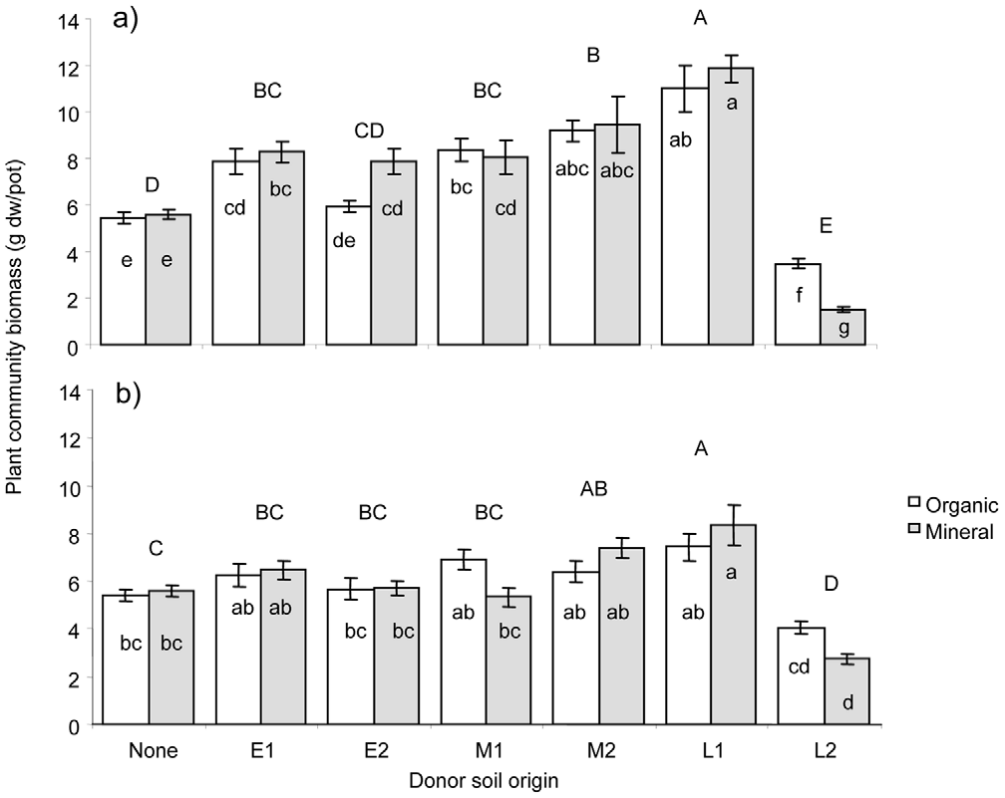
Total plant community biomass in relation to soil treatments. Treatments are arable top soil (organic) or soil from the lower layer (mineral) mixed with a 1∶1 (Fig. 1a) or 1∶5 (Fig. 1b) proportion of donor soil from early (E1 and E2), mid (M1 and M2) or late (L1 and L2) successional restoration grasslands or without donor soil (None). Bars are means ±1 SE, N = 6 for donor soils and N = 12 for ‘none’. Bars not sharing the same letter are significant different at *P*<0.05 with capital letters indicating main effect of donor soil, small case letters indicate effect of donor×receptor soil.

Total plant community biomass (i.e. shoot plus root biomass) was significantly affected by addition of donor soil in a 1∶1 proportion (F_6,77_ = 93.71, P<0.001), whereas the type of receptor soil had no main effect on plant community biomass (F_1,77_ = 0.04, P = 0.85). However, the effect of 1∶1 donor soil depended on whether organic or mineral arable soil was the receptor (donor×receptor soil interaction: F_6,77_ = 6.06, P<0.001) with generally a stronger response to donor soil additions in mineral than in organic receptor soil (Fig. 1a). Addition of donor soil resulted in an overall increased total plant community biomass, especially with donor soil from the later successional sites M2 and L1. However, inoculation of receptor soil with donor soil from the semi-natural field (L2) decreased total plant community biomass, especially in mineral receptor soil (Fig. 1a).

#### Addition of donor soil in a 1∶5 proportion

Total plant community biomass was also affected by donor soil when it was added in smaller proportions (F_6,77_ = 25.15, P<0.001), and again an interaction with the type of receptor soil was found (donor×receptor: F_6,77_ = 3.34, P<0.01) while receptor soil had no main effect (F_1,77_ = 0.56, P = 0.46) (Fig. 1b). The addition of small proportions of donor soil stimulated total plant community biomass in a similar way as large proportions did: especially soil from later successional sites M2 and L1 enhanced plant community biomass while adding soil from the semi-natural field (L2) resulted in reduced plant community biomass, especially in mineral receptor soil (Fig. 1b).

### Comparison between donor soil proportions

**Figure 2:**
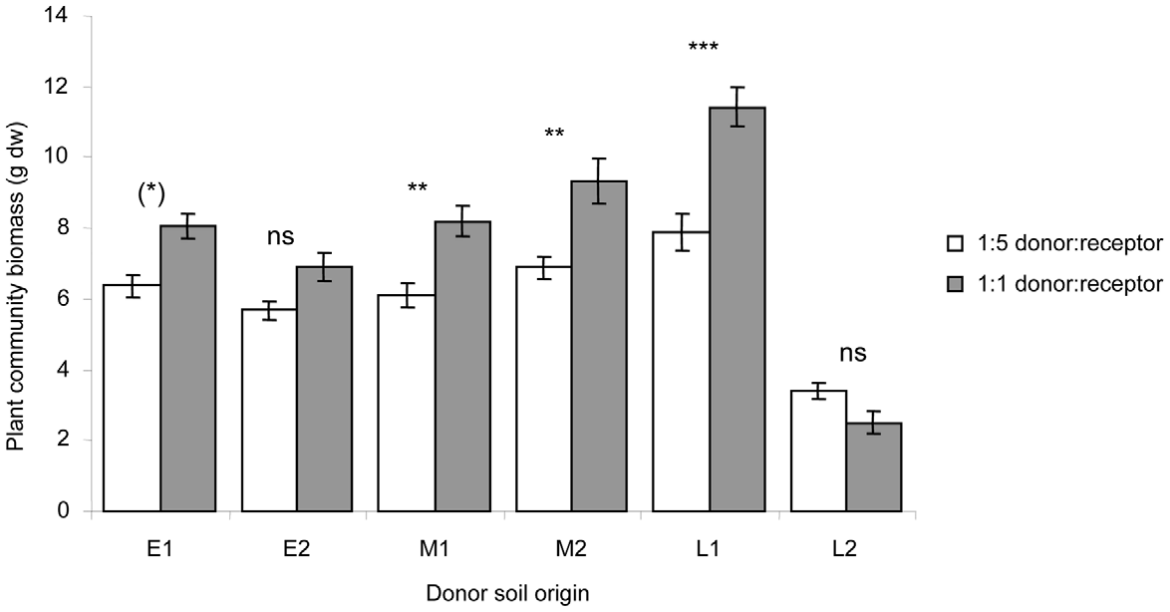
Effect of proportion of donor soil added on total plant community biomass. Soil was mixed in a proportion of 1∶1 or 1∶5 donor∶receptor soil. Significance between proportions within donor soil origin: ns = non significant, (*) *P* = 0.05, ** *P*<0.01, *** *P*<0.001.

Across the treatments that received donor soil the proportion of donor soil addition significantly affected the response of the plant community biomass (F_1,115_ = 50.13, P<0.001), but this effect also depended on the origin of the donor soil (proportion×donor soil interaction: F_5,115_ = 9.69, P<0.001). Large additions of donor soil generally yielded more plant biomass than small additions, especially for additions with the later successional soils M1, M2 and L1 (Fig. 2).

### Effects of donor and receptor soil on plant community diversity

**Figure 3:**
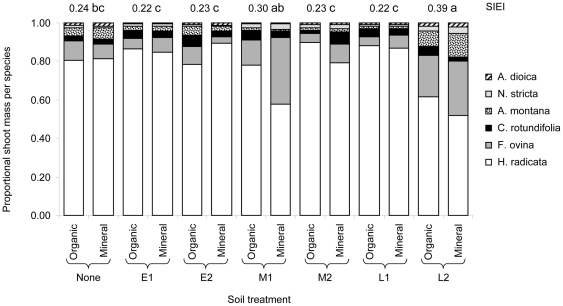
Simpson's evenness index (SIEI) and plant species proportional shoot biomass in response to soil treatments. Numbers above the bars are SIEI values. Treatments as in Fig. 1a. Bars not sharing the same letter are significantly different for SIEI at *P*<0.05 (donor soil main effect).

Donor soil addition in a 1∶1 proportion strongly affected the diversity of the plant communities when considering plant biomass distribution over the different species, illustrated by a significant effect on the Simpson's evenness index (SIEI) (F_6,77_ = 13.80, P<0.0001). This response to donor soil addition did not depend on the type of receptor soil (F_6,77_ = 1.43, P = 0.21) and receptor soil type did not affect the SIEI (F_1,77_ = 2.04, P = 0.16). Plant community evenness was promoted by donor soil from several origins (Fig. 3). Donor soil from the semi-natural grassland L2 strongly promoted the SIEI, and also soil from M1 improved SIEI, albeit to a lesser extend. When less donor soil had been added at a ratio of 1∶5 plant community evenness was not affected by soil addition (F_6,77_ = 1.59, P = 0.16) and ranged from 0.213±0.006 (with E2) to 0.237±0.009 (with L2). In the treatments with the lower donor soil addition SIEI was significantly higher in mineral (0.234±0.005) than in organic (0.222±0.004) receptor soil (F_1,77_ = 5.69, P = 0.019).

### Plant community relations with biotic and abiotic soil properties

**Figure 4:**
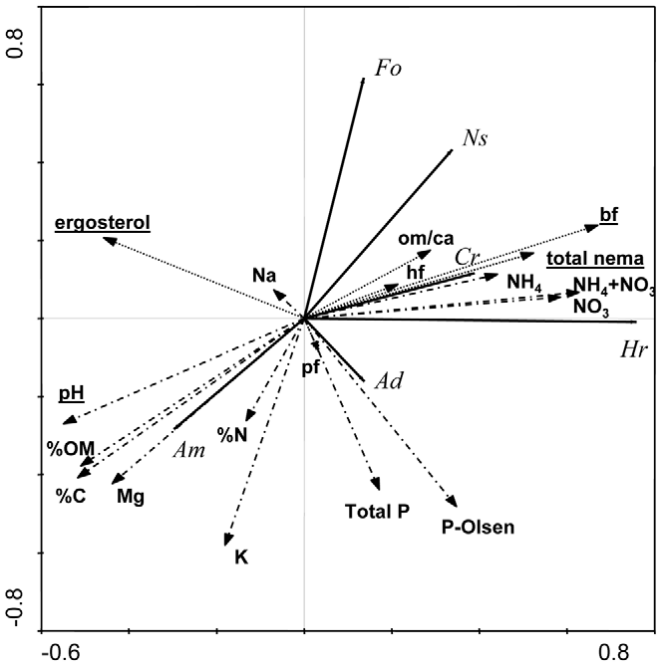
RDA diagram of plant species shoot biomass in relation to initial soil characteristics of the 26 soil treatments (24 mixtures and unmixed mineral and organic arable soil). Fine dotted arrows are abiotic and coarse dotted arrows are biotic characteristics. *Hr* = *Hypochaeris radicata*, *Fo* = *Festuca ovina*, *Cr* = *Campanula rotundifolia*, *Am* = *Arnica montana*, *Ns* = *Nardus stricta*, *Ad* = *Antennaria dioica*. bf = bacterial feeders, pf = plant+root-hair feeders, hf = fungal feeders, ca/om = carnivores+omnivores, total nema = all nematodes.

The variation in the plant communities across all soil treatments could be explained for 56% by our measured set of abiotic and biotic variables, according to multivariate redundancy analysis (RDA) (Fig. 4). The first canonical axis explained as much as 51.6% and the second axis only 1.7% of the total variation. Tests of the significance of specific biotic and abiotic soil variables for plant community composition revealed that only four variables significantly contributed to the canonical axes (underlined variables in Fig. 4). These variables were, in order of diminishing importance: abundance of bacterivorous nematodes (24%, F-ratio = 52.55, P = 0.002), soil ergosterol concentration (23%, F-ratio = 73.63, P = 0.002), total nematode abundance (5%, F-ratio = 16.03, P = 0.002) and soil pH (1%, F-ratio = 3.83, P = 0.036). The RDA diagram also illustrates relations between individual plant species and abiotic and biotic soil properties, as well as relations between these soil properties. A positive relation with mineral nitrogen availability was apparent for *H. radica*, with soil P for *A. dioica*, with soil Mg, %OM and pH for *A. montana* and with nematodes for *C. rotudifolia*. The grass species *F. ovina* and *N. stricta* related negatively to soil P and K. Abundances of nematodes in most of the nematode feeding groups related positively to soil mineral nitrogen availability, while plant-feeding nematodes showed little relation to other factors, except to soil P levels.

### Antennaria dioica response to soil inoculum in the main experiment

**Figure 5:**
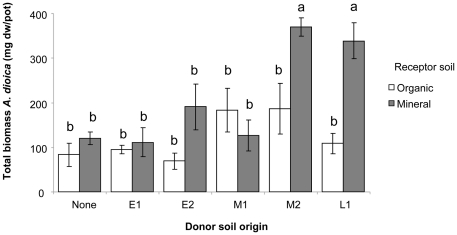
*Antennaria dioica* total dry biomass (mg dw/pot) in relation to soil inoculum from the main experiment.

Total plant biomass production of *A. dioica* was significantly dependent on the composition of the field soil that was inoculated into the sterilised mineral donor soil. There was an effect of the type of donor soil (F_5,55_ = 4.55, P<0.01) and receptor soil (F_1,55_ = 12.19, P<0.001), and the effect of donor soil depended on the type of receptor soil (F_5,55_ = 2.45, P<0.05). Generally *A. dioica* plants were larger when grown with soil inoculum composed of mineral receptor soil and later successional donor soil M2 or L1 (Fig. 5). *Antennaria dioica* biomass was not related to mineral nitrogen availability at the start of the experiment (r = 0.04, p = 0.7).

### Soil fungi in the main experiment

**Figure 6:**
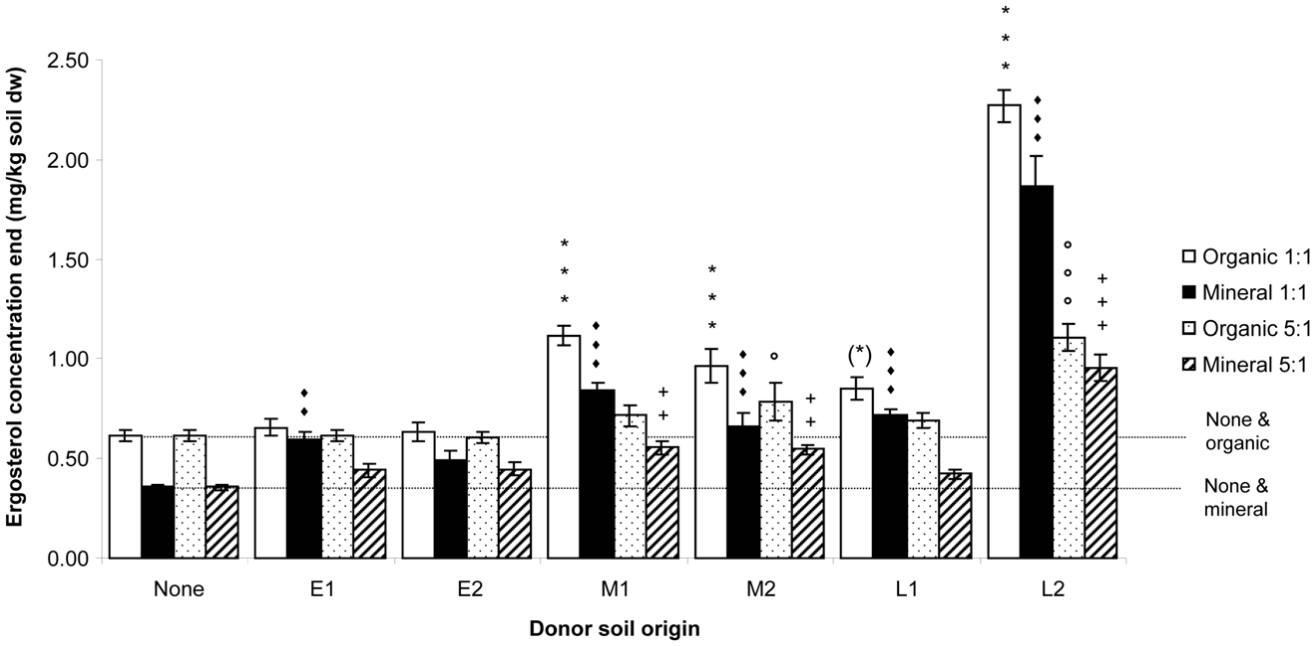
Soil ergosterol concentration (mg/kg soil dw) at the end of the main experiment in relation to the origin and proportion of donor and receptor soil. Significant differences of donor soil addition compared to no addition (none) within receptor soil and its proportion are indicated by * (organic 1∶1), ♦(mineral 1∶1) ° (organic 5∶1), + (mineral 5∶1) with significance P<0.01 for double symbols and P<0.001 for triple symbols and P = 0.06 for (*). Horizontal dotted lines indicate the ergosterol concentrations in the receptor soils without added donor soils.

The ergosterol concentration, a measure of saprophytic soil fungal biomass, at the end of the main experiment was significantly affected by the receptor soil and the donor soil. When added in a large proportion donor soil had a stronger effect than receptor soil (donor F_6,77_ = 174.1, P<0.0001, receptor F_1,77_ = 70.7, P<0.001). However, when added in small proportion the impact of receptor soil was stronger than that of donor soil (donor F_6,77_ = 50.9, P<0.0001, receptor F_1,77_ = 102.8, P<0.0001). Overall soils with organic receptor soil had higher concentrations of ergosterol than soils with mineral receptor soil. Nevertheless the addition of later successional (M and L) donor soil increased ergosterol concentrations as compared to unmixed receptor soil, especially when the donor soil was added in large proportion to mineral receptor soil (Fig. 6).

## Discussion

The restoration of degraded ecosystems may greatly benefit from using an integrated above-belowground approach because of the interdependency of both ecosystem components [18], [32], [33]. Recent studies demonstrate that early and late successional plant species are differentially impacted by feedbacks with soil biota; early successional plant species are reduced and later successional plant species promoted by soil biota [22], [23]. Therefore, in theory late successional plants could be promoted in recently abandoned arable land by combined introduction of target plants and field soil from late successional fields. Yet to date only few studies tried to bring this in practice, probably because of the many open questions that still need to be answered in order to make results of soil additions more predictable [18], [32], [33]. We examined three key questions with respect to the promotion of late successional target plant species in abandoned arable soil by soil inoculation: 1) are donor soil additions more effective after top soil removal, 2) is donor soil origin (with respect to restoration history) of key importance and 3) are the responses to donor soil additions dose dependent?

In answer to our first question, we found that the impact of donor soil addition on plant growth was generally strongest when soil was added to the mineral soil, which becomes exposed following top soil removal. Donor soil addition to the organic top soil was effective as well, but less than in the case of mineral soil. We expected these results based on previous findings that impacts of soil biota are stronger and positive with reduced nutrient availability [28], [30], [31]. The stronger impact of donor soil addition to mineral soil could be due to the rudimentary soil food web in the mineral receptor soil, which may be less competitive towards newly introduced soil biota. In the additional experiment we found confirmation of this idea, because plant growth was stimulated more by adding donor soil from later successional sites to mineral receptor soil than to organic receptor soil. In the field, also some other limitations may need to be controlled in order to further enhance soil biota establishment, for example soil moisture level [34].

In our experiments competition with the seed bank was eliminated by removing spontaneously emerging seedlings, but there were notably fewer weeds in the treatments with mineral than with organic receptor soil. In the field reduced competition with weedy species after top soil removal can provide great benefits to target species [7], [17], yet also after top soil removal restoration of appropriate abiotic conditions is essential in order to make the receptor soil conducive for introductions of desired plants and soil biota [18], [34].

The answer to our second question was positive: the origin of the donor soil was the key factor driving the plant community responses. The largest biomass was produced with soil from late successional grassland and the most even plant community composition with soil from semi-natural grassland. These differences in plant community responses can be explained by especially biotic characteristics of the donor soils, such as ergosterol concentration and abundances of bacterial feeding nematodes. Although we do not have a detailed overview of the soil pathogens and mutualists that can strongly contribute to the responses [22], [23], our results do indicate that differences in soil community composition were at play. Moreover, the ergosterol concentrations at the end of the experiment still depended strongly on the soil treatments suggest that the addition and origin of the donor soil resulted in different biotic communities throughout the experiment. It is noteworthy that depending on soil origin, plant community biomass or evenness was promoted. Our results illustrate the range of possible outcomes depending on interactions between soil biotic and abiotic properties [35]. Soil from semi-natural grassland was extremely poor in phosphorus, which likely incurred larger carbon allocation to mycorrhizal fungi [31]. Such carbon cost may have been disproportionally larger for the dominant species so that it promoted plant community evenness. On the other hand, higher sensitivity to soil pathogens at low phosphorus levels [36], and especially of the dominant species *H. radicata*, remains an alternative explanation. The high abundances of plant-feeding nematodes in the semi-natural field indicate that pathogen pressure may have been relative high in that soil, although we found mostly plant associated/root-hair feeding nematodes which are thought to cause considerable less plant damage than the real parasitic nematodes [44]. The RDA analysis indeed indicates that the abundances of plant-feeding nematodes in the main experiment did not contribute to explain the variability in the biomass of the plant species. Biomass enhancement in soils inoculated with soil from later successional grasslands could be attributed to a soil food web where plant growth promoting biota counterbalanced negative impacts of other biota in the soil inocula.

Finally, we found that the plant community responses depended on the amount of donor soil added. Addition of soil in a 1∶1 proportion to arable receptor soil had a stronger impact on all response variables than adding soil in a proportion of 1∶5. Compared to the control the increase in biomass with L1 donor soil was about 100% with 1∶1 addition and 33% with 1∶5 addition, suggesting that the decline of biomass due to diminishing soil inoculum is not linear with the proportion of soil added. In contrast, the decrease in plant community biomass with L2 was similar for both proportions, while plant community evenness was significantly altered only by large and not by small additions of donor soil. These differential impacts may be attributed to responses caused by different biota that are more or less density dependent in their effects. Responses where rare biota play an important role will then be stronger affected by dose than responses caused by naturally abundant and easy transferable biota [37].

Overall, our work shows that inoculations of later successional soil into ex-arable land can promote the establishment of target plant species, as well as plant community evenness, and this may be most effective after top soil removal. We do recognize that soil from later successional sites is precious and it is not our intention to advocate harming bio-diverse sites for the benefit of restoring degraded sites. Therefore it will be crucial to develop ways of applying the soil introductions such that they are as effective as possible (i.e. needing as little inoculum as possible for maximal success of establishment of target species). The creation of hot spots could be a good approach whereby the precious donor material is introduced locally together with target plants. This approach may require that the inoculum is as intact as possible with as little competition from the residing biota as possible (e.g. after top soil removal). An approach along these lines was recently applied, with success, by Middleton and Bever [23], although the biodiversity of the transplants may decline when the receptor fields are not suitable for taking up the soil biota from the transplants [34].

### Conclusions

Our results contribute to a new perspective for ecosystem restoration management. Current restoration tools tend to be limited to manipulations of soil fertility by top soil removal, grazing and hay making [7], [10] and additions of seeds [15]–[17]. We show that biomass production of late successional plant species on ex-arable land can be promoted profoundly by inoculation with field soil from grasslands of older successional age. We demonstrate that under controlled conditions the origin of donor soil is of greater importance than top soil removal but top soil removal can provide additional benefits. Moreover, responses are dependent on the added dose of donor soil. Now, field tests are needed in order to establish the impact of soil inocula under outdoor conditions, which can be constrained by many more factors that have been controlled in the greenhouse [34].

## Materials and Methods

### Soil origins and properties

All fields that served as source for donor or receptor soil are located on sandy or sandy loam glacial deposits in the central part of the Netherlands (Table 1). All soils were collected end of November 2009. The six grasslands that served as donor soils, were selected from a grassland restoration chronosequence as used by Kardol *et al.* [22] such that they could be grouped into roughly three age categories: E1 and E2 were considered early successional and had been under restoration for 5 years., M1 and M2 were considered mid successional being under restoration between 25 and 30 years, whereas L1 and L2 were designated as late-successional fields with L1 being under restoration since 41 years, and L2 being a semi-natural grassland.

**Table 1:** Codes of field sites, time since abandonment and plant association.

Code	Site		Field age (years)	Lat.	Long.	Plant association^1^
ORG	Arable field near Reijerskamp	organic layer	Arable site	52.02	5.77	Wheat
MIN	Arable field near Reijerskamp	mineral layer	Arable site	52.02	5.77	Wheat
E 1	Oud-Reemst		5	52.04	5.80	16Bc1- Lolio-Cynosuretum
E 2	Reijerskamp		5	52.01	5.78	16Bc1- Lolio-Cynosuretum
M 1	Dennenkamp		28	52.03	5.80	14Bb - Plantagini-Festucion
M 2	Mosselsche Veld		25	52.07	5.74	31Ba1 - Echio-Verbascetum typicum
L 1	Boersbos		41	52.06	6.00	19Aa1 - Galio hercynici-Festucetum ovinae
L 2	Leemputten		Semi-natural site	52.27	5.73	19Aa2 - Gentiano pneumonanthes-Nardetum

Field age = years since abandonment. Lat. = Latitude (°N), Long. = Longitude (°E).

1 According to Schaminée *et al.* [38].

In the arable field top soil was collected from the upper 15 cm layer, and mineral soil from the 50–65 cm layer. This soil was collected from an area of 4×1 m, sieved and homogenized, and top soil and mineral soil were kept separately throughout the further processing. In each of the donor grasslands soil was collected as five randomly distributed turfs of 30×30 cm (l×w) and 15 cm deep. Five random samples were collected from an area of 50×50 m^2^, minimally 20 m from the field edge. Per field site, soil turfs were bulked, sieved (mesh size 1 cm) to remove most of the roots, stones and soil macrofauna, and homogenized. Soil abiotic and biotic parameters were determined on soil subsamples at the start of the experiment (see below and Tables 2, 3, 4, 5).

**Table 2:** Abiotic characteristics of unmixed field soils at the start of the experiment.

Soil	NO_3_(mg.kg^−1^)	NH_4_(mg.kg^−1^)	Olsen-P(mg.kg^−1^)	Total P(mg.kg^−1^)	K(mg.kg^−1^)	Mg(mg.kg^−1^)	Na(mg.kg^−1^)	pH	% OM	Soil texture		
										% sand	% silt	% clay
ORG	6.51	0.17	124	992	88.2	91.6	9.1	6.3	6.4	69.1	29.3	1.6
MIN	5.65	0.00	85	575	78.5	71.9	6.4	6.4	5.5	69.9	28.7	1.4
E1	4.15	0.18	127	1004	87.1	82.3	7.6	6.1	6.0	74.8	23.6	1.6
E2	2.85	0.61	105	781	63.2	57.3	2.4	6.3	4.9	69.5	28.9	1.6
M1	5.33	0.42	35	237	27.7	41.8	9.5	5.5	4.5	84.8	15.1	0.1
M2	4.42	0.48	97	517	28.2	34.1	15.7	5.3	4.7	76.7	22.7	0.6
L1	7.58	0.69	55	207	30.2	19.6	11.8	4.0	5.0	80.6	19.4	0.0
L2	0.00	0.40	0.1	32	34.1	70.5	19.4	5.3	8.4	65.5	33.3	1.2

**Table 3:** Biotic soil characteristics of unmixed field soils at the start of the experiment.

Soil	Ergosterol (mg.kg^−1^)	Nematodes (per 100 g soil dw; n = 2)				
		Total	Bacterial	Plant	Fungal	Omni/Carnivores
ORG	0.80±0.01	1396±181	747±87	496±74	70±22	83±2
MIN	0.45±0.01	682±4	259±27	343±40	48±14	31±2
E1	0.99±0.01	2471±47	1109±26	1017±89	200±7	145±9
E2	0.72±0.01	2277±123	971±84	483±66	184±36	639±63
M1	2.94±0.01	3724±170	2559±163	581±127	218±98	365±37
M2	2.96±0.01	8107±460	5598±95	1673±189	595±69	365±16
L1	1.13±0.01	5908±57	3928±204	1431±48	291±69	258±30
L2	7.99±0.01	5189±58	1897±79	2612±1	525±4	156±18

For nematodes abundances are given for their total and per feeding group (bacterial, plant, fungal feeders, omni- and carnivores).

**Table 4:** Abiotic characteristics of the soils at the start of the experiment.

Soil		Proportion	NO_3_ (mg.kg^−1^)	NH_4_ (mg.kg^−1^)	Olsen-P (mg.kg^−1^)	Total P (mg.kg^−1^)	K (mg.kg^−1^)	Mg (mg.kg^−1^)	Na (mg.kg^−1^)	pH	% OM
receptor	donor	donor∶receptor									
**ORG**	**None**	unmixed	6.51	0.17	124	992	88.2	91.6	9.1	6.3	6.4
**ORG**	**E 1**	1∶5	6.11	0.18	124	994	88.0	90.0	8.9	6.2	6.3
**ORG**	**E 1**	1∶1	5.33	0.18	125	998	87.7	86.9	8.4	6.2	6.2
**ORG**	**E 2**	1∶5	5.90	0.25	120	957	84.0	85.8	8.0	6.3	6.1
**ORG**	**E 2**	1∶1	4.68	0.39	114	887	75.7	74.4	5.8	6.3	5.6
**ORG**	**M 1**	1∶5	6.31	0.22	109	867	78.1	83.3	9.2	6.1	6.1
**ORG**	**M 1**	1∶1	5.92	0.30	79	615	57.9	66.7	9.3	5.9	5.4
**ORG**	**M 2**	1∶5	6.16	0.23	119	913	78.2	82.0	9.9	6.1	6.1
**ORG**	**M 2**	1∶1	5.46	0.33	110	755	58.2	62.8	11.4	5.8	5.5
**ORG**	**L 1**	1∶5	6.69	0.26	112	862	78.5	79.6	9.6	5.9	6.1
**ORG**	**L 1**	1∶1	7.04	0.43	89	601	59.2	55.6	10.5	5.1	5.7
**ORG**	**L 2**	1∶5	5.42	0.21	103	832	79.2	88.0	10.9	6.1	6.7
**ORG**	**L 2**	1∶1	3.25	0.28	62	512	61.1	81.0	14.3	5.8	7.4
**MIN**	**None**	unmixed	5.65	0.00	85	575	78.5	71.9	6.4	6.4	5.5
**MIN**	**E 1**	1∶5	5.40	0.03	92	646	80.0	73.6	6.6	6.3	5.6
**MIN**	**E 1**	1∶1	4.90	0.09	106	789	82.8	77.1	7.0	6.2	5.8
**MIN**	**E 2**	1∶5	5.19	0.10	88	609	76.0	69.5	5.8	6.4	5.4
**MIN**	**E 2**	1∶1	4.25	0.30	95	678	70.8	64.6	4.4	6.3	5.2
**MIN**	**M 1**	1∶5	5.60	0.07	76	519	70.0	66.9	6.9	6.2	5.3
**MIN**	**M 1**	1∶1	5.49	0.21	60	406	53.1	56.8	8.0	5.9	5.0
**MIN**	**M 2**	1∶5	5.45	0.08	87	565	70.1	65.6	7.6	6.2	5.4
**MIN**	**M 2**	1∶1	5.04	0.24	91	546	53.4	53.0	10.1	5.8	5.1
**MIN**	**L 1**	1∶5	5.97	0.12	80	514	70.5	63.2	7.3	6.0	5.4
**MIN**	**L 1**	1∶1	6.62	0.35	70	392	54.4	45.7	9.1	5.2	5.2
**MIN**	**L 2**	1∶5	4.71	0.07	70	484	71.1	71.7	8.6	6.2	6.0
**MIN**	**L 2**	1∶1	2.83	0.20	42	303	56.3	71.2	12.9	5.8	6.9

**Table 5:** Biotic soil characteristics of the soils at the start of the experiment, for nematodes abundances are given for their total and per feeding group (bacterial, plant, fungal feeders, omni- and carnivores).

Soil		Proportion	Ergosterol (mg.kg^−1^)	Nematodes (per 100 g soil dw)				
receptor	donor	donor∶receptor		Total	Bacterial	Plant	Fungal	Omni/Carnivores
**ORG**	**None**	Unmixed	0.80	1502	805	533	74	89
**ORG**	**E 1**	1∶5	0.83	1685	865	623	97	100
**ORG**	**E 1**	1∶1	0.89	2053	986	803	142	121
**ORG**	**E 2**	1∶5	0.78	1664	846	532	95	191
**ORG**	**E 2**	1∶1	0.76	1989	930	529	137	393
**ORG**	**M 1**	1∶5	1.15	1897	1114	545	100	138
**ORG**	**M 1**	1∶1	1.87	2688	1734	569	151	235
**ORG**	**M 2**	1∶5	1.16	2686	1661	740	167	139
**ORG**	**M 2**	1∶1	1.88	5053	3373	1154	353	238
**ORG**	**L 1**	1∶5	0.85	2318	1379	703	115	121
**ORG**	**L 1**	1∶1	0.96	3951	2529	1042	195	185
**ORG**	**L 2**	1∶5	2.00	2120	988	882	150	100
**ORG**	**L 2**	1∶1	4.40	3357	1356	1578	301	123
**MIN**	**None**	Unmixed	0.45	742	282	372	53	34
**MIN**	**E 1**	1∶5	0.54	1052	430	489	79	54
**MIN**	**E 1**	1∶1	0.72	1673	725	722	131	94
**MIN**	**E 2**	1∶5	0.50	1031	411	398	77	145
**MIN**	**E 2**	1∶1	0.59	1609	669	448	126	366
**MIN**	**M 1**	1∶5	0.86	1264	679	411	82	92
**MIN**	**M 1**	1∶1	1.69	2308	1473	488	140	207
**MIN**	**M 2**	1∶5	0.87	2052	1226	606	149	93
**MIN**	**M 2**	1∶1	1.70	4673	3112	1074	342	211
**MIN**	**L 1**	1∶5	0.56	1685	944	569	97	75
**MIN**	**L 1**	1∶1	0.79	3571	2268	962	185	157
**MIN**	**L 2**	1∶5	1.71	1487	553	747	132	54
**MIN**	**L 2**	1∶1	4.22	2976	1094	1497	290	95

### Plants

We planted plant communities consisting of *Antennaria dioica* (L.) Gaertn., *Arnica montana* (L.), *Campanula rotundifolia* (L.), *Hipochaeris radicata* (L.), Festuca ovina* tenuifolia (Sibth.) and *Nardus stricta* (L.), all species that belong to the target plant community *Gentiano pneumonanthes*-*Nardetum* [38] in the main experiment and only *A. dioica* in the additional experiment. Most seeds were provided by a specialized commercial supplier in The Netherlands (Cruydthoeck, Assen), but *Antennaria dioica* and *Arnica montana* were purchased from a specialized supplier in France (B&T world seeds, Aigues-Vives). Seeds were surface sterilized by dipping them in diluted bleach (1% v∶v) for 1–2 minutes and thorough rinsing with demineralised water. Disinfected seeds were germinated on glass beads with demineralised water in a germination cabinet with a day/night regime of 16/8 L/D light at 22/18°C. Seedlings of each species were planted in one of 6 fixed positions per pot and for each of the six replicates a different random plant configuration was used to minimize effects of planting position. Weeds from the soil seed bank were removed during the first 2–3 weeks in order exclude weed competition as a confounding treatment factor. The plant communities were grown in the different soils in pots (21 cm diameter, 15 cm deep) for 4 months in a 16/8 L/D light and a 21/16°C day/night temperature regime and regularly received demineralised water such that a soil moisture level of 16–18% (w∶w) was maintained.

### Experimental design

#### Main experiment

The growth response of mixed plant communities to donor soil additions in arable soil was tested in a greenhouse experiment. The soil treatments consisted of field soil from an arable field, denominated as “receptor soil”, inoculated with soil from six grasslands that have been restored for a variety of years, denominated as “donor soils”. The arable soil was taken from the top (organic) or lower (mineral) soil layer as to test the effects of soil inoculation in fields without or with top soil removal, respectively. We inoculated the organic and mineral arable field soil with a small (1∶5) or large (1∶1) amount of donor soils (proportions based on dry weights) from one of six grasslands of different age along a restoration chronosequence, and we included unmixed receptor soils as controls. This resulted in 26 soil treatments (2 types of receptor soil×6 donor soil origins×2 proportions of donor soil = 24+2 receptor soil types without donor soil), which were replicated in six randomized blocks, resulting in 168 experimental units.

#### Additional experiment

In order to test the impact on plant growth of the soil biota in the soils used in the main experiment an additional experiment was set up in parallel, with the soils of the 1∶1 mixtures and the unmixed receptor soils from the main experiment as inoculum. We used a similar approach as Kardol *et al.* [22]. Plants were grown in soil consisting of 5/6 parts bulk sterilised mineral field soil and 1/6 parts field soil inoculum. The bulk sterilised soil was arable receptor soil collected after top soil removal which was sterilised by γ-irradiation (25 kGy). The field soil inoculum consisted of the soil of the different mixtures and the unmixed receptor soil as used in the main experiment. Due to the limited availability of L2 receptor soil, soils that contained L2 donor soil were not included. This resulted in 12 treatments, replicated in six blocks, of which two of the treatments contained 1/6 receptor field soil (organic or mineral) and the other treatments contained 1/12 donor field soil (of E1, E2, M1, M2 or L1) and 1/12 receptor field soil (organic or mineral). The rest (5/6 parts) of the soil in each treatment was sterilised mineral receptor soil. To test plant growth response to the soil treatments single individuals of the same plant species were used, in order to avoid plant competition effects interfering with responses of the focal species to the soil biota. As focal plant species *A. dioica* was chosen because it is in decline (red list species) and because it was not the dominant species in the plant communities in the main experiment. A single seedling of the target plant species *Antennaria dioica* was planted in each of the 72 containers filled with 300 g of the soil mixtures with a moisture content of 20%(w∶w). Seedlings were planted three weeks after their germination and harvested after ten weeks of growth. The plants were grown in a greenhouse under the same controlled conditions as the plant communities of the main experiment.

### Measurements

#### Plants

After 4 months of growth in the main experiment and 2.5 months of growth in the additional experiment, shoots and roots of each plant species were collected, dried to constant weight at 70°C and weighed. The reported total biomass comprises shoot plus root biomass. From each pot of the main experiment, a 300 g soil subsample was collected for the analysis of soil abiotic and biotic characteristics.

#### Abiotic soil characteristics

Soil mineral content was determined using sieved (4 mm mesh) fresh soil of all 8 unmixed field soils before the experiment and of all 168 pots at the end of the main experiment. Mineral N was extracted from soil subsamples (10 g dry weight eq.) by shaking in 50 ml 1 M KCl for 2 h, and filtering through a Whatman filter. The concentrations of NH_4_ and NO_3_ in the filtrate were determined colorimetrically using Traacs 800 auto-analyzer (TechniCon Systems, Inc.). Available phosphorus (P-Olsen) was extracted using a 0.5 M solution of NaHCO_3_ at pH 8.5 and determined according to Olsen and Sommers [39] and concentrations of K, Na and Mg were determined after CaCl_2_ extraction [40]. Total soil N and P content were determined by digestion with a mixture of H_2_SO_4_-Se and salicylic acid [41]. Soil organic matter (OM) content was determined via loss on ignition of dry soil burned at 430°C as a percentage of total weight. Soil %C and %N in oven-dry soil was determined using an elemental analyser (Eager EA1112, Interscience, Breda). Soil water content was determined gravimetrically from fresh and oven-dry (105°C) soil and pH of fresh soil was measured in 1∶2.5 (dry weight) soil∶water suspensions. Soil texture was determined using soil particle sizes distributions of freeze dried, sieved (1 mm mesh) soil, measured by laser diffraction with a Malvern 2000 particle size analyzer (Malvern Instruments Ltd, Malvern, UK). The proportion of mineral particles <2 µm were assigned to the clay fraction, particles of 2–50 µm to the loam and 60–1000 µm to the sand fraction.

#### Biotic soil characteristics

Ergosterol, as a measure of soil fungal biomass [42], was extracted from soil at the start and at the end of the main experiment and quantified by HPLC analysis by standard procedures [26]. Nematodes were extracted from 100 cm^3^ of fresh soil by Oostenbrink elutriators [43], extracts were poured on a double cotton wool filter (Hygia milac filter, Hartmann BV, Nijmegen, the Netherlands), put on a tray with 100 ml water from which nematodes were collected after 24 hours incubation at 20°C and concentrated into 10 ml volume. All nematodes in 2 ml subsamples were examined using a reverse-light microscope (×100–400), counted and classified into feeding-groups according to Yeates *et al.* [44] as bacterial feeders, plant feeders (including plant associated nematodes), fungal feeders and omni/carnivores.

### Data analysis

Plant evenness in the main experiment was calculated as Simpson's evenness index SIEI which equals 1/Σ p_i_^2^×1/S, where p_i_ represents the proportional contribution of shoot biomass of species i to the total plant community shoot biomass and S is the number of species in the community [45]. Soil characteristics of the 26 soil treatments at the start of the main experiment were based on the data of the unmixed soils and the proportion in which they were mixed. To test effects of receptor soil type and of donor soil origin and their interaction in the main and in the additional experiment two-way Analysis of Variance (ANOVA) was performed with type of arable receptor soil (organic or mineral), donor soil origin (one of the six grasslands or no addition) and receptor×donor soil as fixed factors and block as a random factor. In the main experiment two-way ANOVA was applied separately for the dataset comprising the small (1∶5) and large (1∶1) additions of donor soil and for the dataset with soil of the main experiment as inoculum for sterilised soil. The effects of the proportion of added donor soil were tested on the dataset comprising all treatments with donor soil addition, but excluding the treatments with unmixed receptor soils, by means of ANOVA with donor soil, receptor soil, proportion of donor soil and their interactions as fixed and block as random factors. Differences between the treatments were tested using Tukey's posthoc tests (for Unequal N in cases of unequal number of replicates) or LSD test (for *A. dioica* mass in the additional experiment). Homogeneity of variances was verified using Levene's test and biomass, SIEI values and ergosterol end concentrations were sqrt transformed to achieve homoscedasticity. ANOVAs were performed using STATISTICA (release 9.0, Statsoft, Inc.). Relations between plant species biomass and initial soil abiotic and biotic characteristics of the 26 soil treatments (24 mixtures and unmixed mineral and organic arable soil) in the main experiment were analysed by multivariate redundancy analysis (RDA) and Monte Carlo permutation tests (499 unrestricted permutations) using CANOCO, version 4.5 [46].
